# Supplementary material for: Soybean Thrips (Thysanoptera: Thripidae) Harbor Highly Diverse Populations of Arthropod, Fungal and Plant Viruses
Source: Viruses. 2020 Dec 1;12(12):1376. doi: 10.3390/v12121376 (PMC7761488; doi:10.3390/v12121376)
Supplement: Supplementary file 1 [file viruses-12-01376-s001.pdf]

Supplementary Table 1. Virus-like sequences assembled from soybean thrips RNA-seq data

Sequence Name	Accession Number	Longest Contig	Number of Reads	Percent Coverage	Mean Coverage Depth	Best Match (Accession Number)	Percent aa Identity
**Positive-sense RNA viruses**							
**Order: *Picornavirales***							
**Iflavirus-like**							
*Hubei arthropod virus 1	MT293153	9635	834,888	100%	19,443	Hubei arthropod virus 1 (YP_009336629.1)	96.5%
Soybean thrips iflavirus 1	MT195546	10806	79,433	100%	1,742	Hubei picorna-like virus 32 (YP_009337306.1)	48.7%
Soybean thrips iflavirus 2	MT195547	10286	2.34×10^6	100%	38,908	Ixodes scapularis iflavirus (BBD75427.1)	24.9%
Soybean thrips iflavirus 3	MT195548	10256	60,991	100%	1,425	Hubei picorna-like virus 28 (YP_009337284.1)	45.4%
Soybean thrips iflavirus 4	MT224138	10073	13,905	100%	327	La Jolla virus (AKH40286.1)	36.2%
Soybean thrips iflavirus 5	MT224139	9900	66,312	99%	1,586	Vespa velutina associated ifla-like virus (QGL51726.1)	51.5%
Soybean thrips iflavirus 6	MT224140	9488	6,855	96%	164	Pityohyphantes rubrofasciatus iflavirus (YP_009351892.1)	37.0%
Soybean thrips iflavirus 7	MW023868	8951	49,369	100%	1,143	Sacbrood virus (AJA38041.1)	28.1%
Soybean thrips iflavirus 8	MT240781	8886	743,592	99%	19,994	Hubei picorna-like virus 41 (YP_009337750.1)	51.4%
Soybean thrips iflavirus 9	MT224141	8391	49,369	92%	28	Darwin bee virus 2 (AWK77843.1)	42.8%
Soybean thrips ifla-like virus 10	MT224142	7343	2,852	80%	74	Darwin bee virus 2 (AWK77843.1)	70.1%
Soybean thrips iflavirus 11	MW039357	1381	117	16%	3	Harmonia axyridis virus 1 (QFR15912.1)	51.6%
Soybean thrips picorna-like virus 5	MW039359	1425	102	14%	2	Spodoptera exigua iflavirus 1 (YP_004935363.1)	42.0%
**Dicistrovirus-like**							
Soybean thrips dicistrovirus 1	MT195550	10079	4,541	100%	106	Hubei picorna-like virus 14 (YP_009337313.1)	61.6%
Soybean thrips dicistrovirus 2	MT224137	9847	16,016	100%	363	Griffin dicistrovirus (AOC55056.1)	28.5%
Soybean thrips picorna-like virus 1	MT240797	8658	1,997	100%	55	Pteromalus puparum small RNA-containing virus (ACG60668.1)	65.3%
Soybean thrips picorna-like virus 3	MT240798	5849	800	59%	19	Wuhan arthropod virus 2 (YP_009342286.1)	44.5%
Soybean thrips picorna-like virus 4	MT240799	4035	397	38%	9	Myrmica scabrinodis virus 2 (AYW51544.1)	59.8%
Soybean thrips picorna-like virus 10	MW023863	6896	28,677	100%	959	Melipona quadrifasciata virus (QCE20587.1)	39.4%
*Aphid lethal paralysis virus	MT240796	9883	89,940	100%	2,166	Aphid lethal paralysis virus (AGF84786.1)	98.9%
*Rhopalosiphum padi virus	MW039360	1848	297	38%	1	Rhopalosiphum padi virus (AWM98375.1)	96.9%
*Rhopalosiphum padi virus	MW039361	1779	244	18%	4	Rhopalosiphum padi virus (AWM98375.1)	88.3%
**Calicivirus-like**							
*Soybean thrips Pernambuco virus	MT293133	5160	726	100%	32	Pernambuco virus (QCP68850.1)	95.3%
**Other Picornavirus-like**							
Soybean thrips picorna-like virus 2	MW039358	1828	136	31%	5	Insect-associated waikavirus 1 (QHB15178.1)	36.2%
Soybean thrips picorna-like virus 6	MT293127	3335	180	84%	11	Volivirus sp. (APO40851.1)	65.8%
Soybean thrips picorna-like virus 7	MT293128	8960	4,385	97%	114	Perth bee virus 3 (AWK77896.1)	62.6%
Soybean thrips picorna-like virus 8	MT293129	4220	2,156	41%	7	Lampyris noctiluca iflavirus 1 (QBP37019.1)	35.0%
Soybean thrips picorna-like virus 9	MT293152	3007	13,400	33%	8	Thika virus (YP_009140561.1)	50.8%
Soybean thrips picorna-like virus 11	MW023864	6617	47,002	79%	1,304	Solenopsis invicta virus 13 (QBL75905.1)	30.0%
**Bicistroinc Picornavirus-like**							
Soybean thrips bicistronic virus 1	MT195549	8654	12,928	97%	351	Hubei picorna-like virus 51 (YP_009337724.1)	50.4%
*Hubei picorna-like virus 55	MT293126	9802	16,146	100%	379	Hubei picorna-like virus 55 (YP_009337269.1)	99.0%
**Order: *Sobelivirales* (Sobemovirus-like)**							
Soybean thrips sobemo-like virus 1	MT293131	3592	10,363	100%	645	Hubei tetragnatha maxillosa virus 6 (YP_009330269.1)	32.1%
Soybean thrips sobemo-like virus 2	MT293132	3272	639	100%	45	Wenzhou sobemo-like virus 2 (YP_009337335.1)	40.6%
Soybean thrips sobemo-like virus 3	MW023865	2734	2,983	100%	250	Mlepnos solemo-like virus (QIJ70120.1)	32.8%
Soybean thrips sobemo-like virus 4	MW023866	3407	116,224	100%	7,759	Hubei sobemo-like virus 25(YP_009330120.1)	67.6%
Soybean thrips sobemo-like virus 5	MW023867	3401	14,787	99%	983	Hubei sobemo-like virus 35 (YP_009330090.1)	61.4%
Soybean thrips sobemo-like virus 6	MW039352	2744	3,701	96%	289	Wuhan house centipede virus 4 (YP_009342451.1)	31.0%

Soybean thrips sobemo-like virus 7	MW039353	2704	5,908	93%	429	Wuhan arthropod virus 4 (YP_009342307.1)	54.0%
Soybean thrips sobemo-like virus 8	MW039354	2671	2,233	100%	195	Hubei sobemo-like virus 7 (YP_009330005.1)	42.5%
Soybean thrips sobemo-like virus 9	MW039355	2431	141	87%	12	Hubei sobemo-like virus 9 (YP_009329983.1)	62.8%
Soybean thrips sobemo-like virus 10	MW023863	3085	11,214	100%	830	Hubei sobemo-like virus 42 (YP_009330107.1)	54.5%
# Soybean thrips sobemo-like virus 11	MW023864	2639	171	42%	6	Sesbania mosaic virus (NP_066393.4)	60.0%
*# Lucerne transient streak virus	MT224146	4227	1,963	99%	107	Lucerne transient streak virus (YP_007438849.1)	96.7%
**Order: *Martellivirales* (Virgavirus-like)**							
Soybean thrips virga-like virus 1	MT240784	10947	14,895	94%	290	Beult virus (AWA82276.1)	31.6%
Soybean thrips virga-like virus 2	MT240785	3339	169	32%	4	Hubei virga-like virus 11 (QIJ70141.1)	62.7%
Soybean thrips nege-like virus 1	MT240782	9369	73,252	97%	485	San Bernardo virus (AQM55436.1)	45.6%
Soybean thrips nege-like virus 2	MW039375	2003	73	19%	2	Wuhan house centipede virus 1 (YP_009342435.1)	45.1%
Soybean thrips nege-like virus 2	MW039377	1289	82	13%	2	Wuhan house centipede virus 1 (YP_009342435.1)	28.9%
#Soybean thrips iivi-like virus 1, RNA1	MT240787	3929	2,273	95%	127	Grapevine associated jivivirus 2 (QIJ25701.1)	33.9%
#Soybean thrips iivi-like virus 1, RNA2	MW039368	3245	636	100%	44	Grapevine associated jivivirus 2 (QIJ25702.1)	55.1%
#Soybean thrips iivi-like virus 2, RNA1	MT240786	3733	533	90%	28	Grapevine associated jivivirus 2 (QIJ25701.1)	44.1%
#Soybean thrips iivi-like virus 2, RNA2	MW039369	1756	243	55%	18	Grapevine associated jivivirus 1 (QIJ25699.1)	65.0%
*# Peanut stunt virus, RNA1	MT293135	3106	570	93%	40	Peanut stunt virus (NP_049331.1)	98.5%
*# Peanut stunt virus, RNA2	MT293136	2621	262	89%	21	Peanut stunt virus (NP_049332.1)	94.8%
*# Peanut stunt virus, RNA3	MT293137	2026	2,141	93%	231	Peanut stunt virus (NP_049333.1)	88.9%
*# Turnip vein-clearing virus	MT293141	3682	444	58%	10	Turnip vein-clearing virus (NP_046151.1)	97.2%
**Order: *Tolivirales* (Tombusvirus-like)**							
Soybean thrips tombus-like virus 1	MT240788	4172	1,393	100%	74	Sanxia tombus-like virus 5 (YP_009337188.1)	35.3%
Soybean thrips tombus-like virus 2	MT240789	4300	20,789	100%	1,129	Sanxia tombus-like virus 5 (YP_009337188.1)	39.9%
Soybean thrips tombus-like virus 3	MT240790	4088	2,275	100%	127	Wuhan spider virus 9 (YP_009344999.1)	43.5%
Soybean thrips tombus-like virus 4	MT240791	3818	5,351	94%	292	Hubei tombus-like virus 18 (YP_009336696.1)	41.4%
Soybean thrips tombus-like virus 5	MT240793	3783	4,638	100%	263	Wuhan spider virus 9 (AOC55070.1)	40.7%
# Soybean thrips tombus-like virus 6	MT240794	3399	687	84%	36	Japanese iris necrotic ring virus (AFL55711.1)	39.0%
Soybean thrips tombus-like virus 7	MW023842	2823	394	60%	19	Vespa velutina associated permutotetra-like virus 2 (QGL51737.1)	30.2%
Soybean thrips tombus-like virus 8	MW023843	2325	373	57%	21	Wuhan spider virus 9 (YP_009344999.1)	37.2%
Soybean thrips tombus-like virus 9	MW023844	1946	140	48%	8	Wuhan spider virus 9 (YP_009344999.1)	36.1%
Soybean thrips tombus-like virus 10	MW023845	1796	307	81%	32	Cushing virus (QED21532.1)	47.7%
Soybean thrips tombus-like virus 11	MW023846	1418	99	26%	4	Hubei tombus-like virus 2 (YP_009336967.1)	74.4%
*Wuhan insect virus 21, RNA1	MT240792	3733	3,201	100%	182	Wuhan insect virus 21 (BBV14765.1)	94.6%
*Hubei tombus-like virus 2	MW039367	1381	70	25%	2	Hubei tombus-like virus 2 (YP_009336967.1)	99.3%
**Order: *Amarillovirales* (Flavivirus-like)**							
Soybean thrips virus 1, segment 1	MW023851	3290	9,296	100%	651	Wuhan flea virus (YP_009179403.1)	45.5%
Soybean thrips virus 1, segment 3	MW023852	2631	7,605	92%	614	Wuhan flea virus (YP_009179404.1)	39.1%
Soybean thrips virus 1, segment 4	MW023853	2730	21,324	96%	1,720	Wuhan flea virus (YP_009179399.1)	23.7%
Soybean thrips virus 2, segment 1	MW023854	3096	2,257	100%	164	Wuhan aphid virus 2 (YP_009179378.1)	44.0%
Soybean thrips virus 2, segment 2	MW023855	2131	7,423	98%	777	Wuhan aphid virus 2 (YP_009179385.1)	25.4%
Soybean thrips virus 2, segment 3	MW023856	3071	271,883	100%	20,058	Wuhan aphid virus 2 (YP_009179379.1)	32.0%
Soybean thrips virus 2, segment 4	MW023857	2819	21,952	99%	1,792	Wuhan aphid virus 2 (YP_009179387.1)	55.9%
Soybean thrips virus 3, segment 1	MW033628	3061	17,735	100%	1,355	Wuhan aphid virus 2 (YP_009179378.1)	75.9%
Soybean thrips virus 3, segment 2	MW033629	2120	4,832	99%	516	Wuhan aphid virus 2 (QDF44110.1)	54.1%
Soybean thrips virus 3, segment 3	MW033630	1814	14,470	67%	1,793	Wuhan aphid virus 2 (QDF44111.1)	26.7%
Soybean thrips virus 3, segment 4	MW033631	3228	23,892	100%	1,881	Wuhan aphid virus 2 (YP_009179387.1)	24.3%
Soybean thrips virus 4, segment 1	MW033624	3279	262,587	100%	17,893	Wuhan aphid virus 1 (YP_009179388.1)	40.8%

Soybean thrips virus 4, segment 3	MW033625	2804	41,928	100%	3,440	Wuhan aphid virus 2 (YP_009179379.1)	61.8%
Soybean thrips virus 4, segment 4	MW033626	3002	290,376	100%	21,463	Wuhan aphid virus 2 (YP_009179386.1)	25.2%
Soybean thrips virus 5, segment 4	MW033632	2699	568	95%	47	Wuhan aphid virus 2 (YP_009179387.1)	49.4%
*Wuhan aphid virus 1, segment 1	MW023847	3154	8,558	100%	620	Wuhan aphid virus 1 (YP_009179388.1)	86.1%
*Wuhan aphid virus 1, segment 2	MW023848	2171	1,962	100%	207	Wuhan aphid virus 1 (YP_009179381.1)	78.9%
*Wuhan aphid virus 1, segment 3	MW023849	2840	2,159	100%	175	Wuhan aphid virus 1 (YP_009179389.1)	85.4%
*Wuhan aphid virus 1, segment 4	MW023850	2834	15,315	100%	1,270	Wuhan aphid virus 1 (YP_009179383.1)	79.6%
*Shayang fly virus 4	MW039350	2101	228	23%	6	Shayang fly virus 4 (YP_009179225.1)	84.8%
**Order: *Tymovirales* (Tymovirus-like)**							
*Hubei macula-like virus 3	MT240795	6024	2,649	100%	64	Hubei macula-like virus 3 (APG77727.1)	93.0%
# Soybean carlavirus 1	MT293130	8631	3,212	100%	87	Red clover carlavirus A (AUF71590.1)	62.0%
*# Red clover vein mosaic virus	MT293134	8482	2,109	99%	56	Red clover mosaic virus (YP_002647021.1)	97.2%
**Order: None (Permutotetravirus-like)**							
*Aphis glycines virus 2	MW033624	4807	9,499	99%	9,499	Aphis glycines virus 2 (YP_009179352.1)	99.8%
Soybean thrips permutotetra-like virus 1	MT240780	4290	251,668	100%	13,329	Defiwi virus (AOC55067.1)	34.0%
Soybean thrips permutotetra-like virus 2	MW039362	1794	332	38%	16	Vespa velutina associated (QGL51737.1)	38.2%
Soybean thrips permutotetra-like virus 3	MW039363	1514	76	31%	3	Hubei permutotetra-like virus 7 (YP_009337365.1)	38.4%
**Order: *Wolframvirales* (Narnavirus-like)**							
Soybean thrips narna-like virus 1	MT293142	2785	5,400	97%	368	Hubei narna-like virus 15 (YP_009337783.1)	40.5%
Soybean thrips narna-like virus 2	MT293143	2481	2,098	94%	175	Barley aphid RNA virus 6 (BBV14771.1)	34.4%
**Order: *Cryppavirales* (Mitovirus-like)**							
Soybean thrips mito-like virus 1	MT293144	2377	4,567	100%	432	Mitovirus sp. (QDH89786.1)	44.2%
Soybean thrips mito-like virus 2	MT293145	2630	226	94%	17	Plasmopara viticola associated mitovirus 56 (QIR30279.1)	46.2%
**Order: *Ourlivirales* (Ourmiavirus-like)**							
Soybean thrips ourmia-like virus 1A	MW039364	2237	214	96%	17	Plasmopara viticola associated ourmia-like virus 72 (QGY72602.1)	77.7%
Soybean thrips ourmia-like virus 1B	MW039365	2174	170	93%	13	Plasmopara viticola associated ourmia-like virus 72 (QGY72602.1)	78.1%
Soybean thrips ourmia-like virus 2	MW039366	1702	43	78%	0.6	Plasmopara viticola associated ourmia-like virus 78 (QGY72608.1)	62.9%
**Negative-stranded RNA viruses**							
**Order: *Articulavirales* (Quaranjavirus-like, Thogotovirus-like)**							
Soybean thrips quaranja-like virus 1, segment 1	MW033634	2421	745	99%	76	Old quarry swamp virus (AYP67574.1)	34.6%
Soybean thrips quaranja-like virus 1, segment 2	MW033635	2345	408	96%	42	Old quarry swamp virus (AYP67573.1)	49.5%
Soybean thrips quaranja-like virus 1, segment 3	MW033636	2274	588	99%	64	Old quarry swamp virus (AYP67572.1)	26.6%
Soybean thrips quaranja-like virus 1, segment 5	MW033637	1813	302	100%	42	Old quarry swamp virus (AYP67576.1)	27.5%
Soybean thrips quaranja-like virus 2, segment 2	MW033638	2434	429	99%	44	Mason Creek virus (QFQ60710.1)	65.0%
Soybean thrips quaranja-like virus 2, segment 3	MW033639	2362	524	98%	54	Mason Creek virus (QFQ60711.1)	52.5%
Soybean thrips quaranja-like virus 2, segment 4	MW033640	1608	228	98%	32	Mason Creek virus (QFQ60713.1)	48.1%
Soybean thrips quaranja-like virus 3, segment 2	MW033641	2459	304	100%	28	Wuhan Mosquito Virus 7 (AJG39095.1)	45.5%
Soybean thrips quaranja-like virus 4, segment 3	MW033642	2188	494	96%	46	Old quarry swamp virus (AYP67572.1)	36.8%
Soybean thrips thogotovirus 1, segment 1	MT195539	2413	12,438	100%	1,179	Hubei orthoptera virus 6 (APG77907.1)	37.9%
Soybean thrips thogotovirus 1, segment 2	MT195540	2214	17,229	100%	1,795	Hubei orthoptera virus 6 (APG77906.1)	53.6%
Soybean thrips thogotovirus 1, segment 3	MT195541	2014	34,764	100%	4,041	Hubei orthoptera virus 6 (APG77908.1)	44.6%
Soybean thrips thogotovirus 1, segment 4	MT195542	1646	6,403	100%	884	Hubei orthoptera virus 6 (APG77909.1)	33.3%
Soybean thrips thogotovirus 1, segment 5	MT195543	1588	22,071	100%	3,147	Hubei orthoptera virus 6 (APG77910.1)	35.5%
Soybean thrips thogotovirus 2, segment 4	MW033633	1540	94	71%	10	Dhori thogotovirus (YP_009352874.1)	34.6%

**Order: *Bunyavirales* (Bunyavirus-like)**							
Soybean thrips bunya-like virus 1	MT224143	9159	10,758	100%	266	Bronnoya virus (ASY03250.1)	29.2%
Soybean thrips bunya-like virus 2	MW033651	2917	193	42%	6	Hubei bunya-like virus 8 (APG79291.1)	43.5%
Soybean thrips bunya-like virus 3	MT224145	6585	961	100%	33	Ganda bee virus (YP_009666981.1)	46.9%
Soybean thrips bunya-like virus 4	MT293149	4329	15,588	94%	775	Geran virus (AKC89341.1)	27.9%
Soybean thrips bunya-like virus 5	MW023862	4684	4,560	100%	218	Ixodes scapularis (BBD75426.1)	24.7%
Soybean thrips bunya-like virus 6	MW033651	2562	99	38%	3	Wuhan insect virus 16 (APG79216.1)	63.8%
Soybean thrips bunya-like virus 7	MW033652	1469	55	23%	2	Cumuto virus (YP_009664615.1)	36.3%
Soybean thrips bunya-like virus 8	MW033653	1538	59	47%	4	Yichang Insect virus (YP_009305144.1)	29.1%
Soybean thrips bunya-like virus 9	MW033654	2451	9,058	66%	554	Norway nairovirus 1 (ASY03237.1)	27.5%
Soybean thrips bunya-like virus 9	MW033655	1538	174	28%	5	South Bay virus (ANT80543.1)	27.8%
Soybean thrips negative-stranded RNA virus 2, segment L	MT224152	9002	4,151	91%	92	Hubei myriapoda virus 5 (YP_009345128.1)	33.8%
Soybean thrips negative-stranded RNA virus 2, segment S	MW033657	2426	1,944	94%	169	Hubei myriapoda virus 5 (YP_009345127.1)	23.7%
+ Soybean thrips associated tenui-like virus 1, segment 1	MT224144	8381	28,519	93%	746	Rice stripe tenuivirus (ACG58336.1)	29.7%
+ Soybean thrips associated tenui-like virus 1, segment 4	MW033650	2421	4,885	100%	459	Rice hoja blanca tenuivirus (YP_009449444.1)	27.1%
+ Soybean thrips associated orthotospovirus 1, segment L	MT195544	8917	31,857	100%	818	Capsicum chlorosis virus (AJD14704.1)	32.0%
+ Soybean thrips associated orthotospovirus 1, segment M	MT195545	4965	24,362	100%	1,180	Calla lily chlorotic spot virus (ACO52398.1)	29.9%
*+ Soybean vein necrosis orthotospovirus, segment L	MT293138	9038	130,169	100%	3,273	Soybean vein necrosis virus (ADX01591.1)	99.5%
*+ Soybean vein necrosis orthotospovirus, segment M	MT293139	4941	49,083	100%	2,259	Soybean vein necrosis virus (AMB72701.1)	98.7%
*+ Soybean vein necrosis orthotospovirus, segment S	MT293140	2622	24,466	100%	2,150	Soybean vein necrosis virus (AMB72703.1)	100.0%
*+ Tomato spotted wilt orthotospovirus, segment S	MW033649	1575	2,026	52%	59	Tomato spotted wilt orthotospovirus (APG79521.1)	98.7%
**Order: *Jingchuvirales* (Chuvirus-like)**							
Soybean thrips chu-like virus 1, Segment L	MT224150	7386	5,064	100%	104	Hubei chuvirus-like virus 1 (YP_009337904.1)	33.3%
Soybean thrips chu-like virus 1, Segment S	MT293148	2272	96	57%	5	Hubei chuvirus-like virus 1 (YP_009337905.1)	32.4%
Soybean thrips chu-like virus 2	MT293147	4327	967	90%	44	Wuchang Cockroach Virus 3 (YP_009666257.1)	31.6%
Soybean thrips chu-like virus 3	MT293151	2020	1,279	57%	23	Imjin River virus 1 (YP_009182178.1)	28.6%
Soybean thrips chu-like virus 4	MW033643	2603	340	23%	7	Hubei chuvirus-like virus 3 (YP_009337090.1)	31.3%
Soybean thrips chu-like virus 5	MW033644	2369	362	22%	7	Guangdong red-banded snake chuvirus-like virus (AVM87273.1)	28.6%
Soybean thrips chu-like virus 6	MW033645	1501	163	43%	8	Wuchang Cockroach Virus 3 (YP_009666257.1)	28.9%
Soybean thrips chu-like virus 7	MW033646	1275	65	11%	1	Blacklegged tick chuvirus-2 (AUW34383.1)	44.0%
Soybean thrips chu-like virus 8	MW033647	1755	55	44%	3	Hubei chuvirus-like virus 1 (YP_009337905.1)	30.4%
**Order: *Serpentovirales***							
Soybean thrips negative-stranded RNA virus 1	MT224151	8927	5,517	100%	146	Cladosporium cladosporioides negative-stranded RNA virus 1 (QDB75017.1)	29.7%
Soybean thrips negative-stranded RNA virus 3	MT224153	8937	6,342	100%	169	Cladosporium cladosporioides negative-stranded RNA virus 1 (QDB75017.1)	28.4%
Soybean thrips negative-stranded RNA virus 6	MT293150	4807	375	62%	11	Cladosporium cladosporioides negative-stranded RNA virus 1 (QDB75017.1)	30.6%
Soybean thrips negative-stranded RNA virus 7	MW039378	8923	3,159	100%	84	Cladosporium cladosporioides negative-stranded RNA virus 1 (QDB75017.1)	28.8%
Soybean thrips negative-stranded RNA virus 8	MW039379	2111	197	29%	6	Rhizoctonia solani negative-stranded virus 2 (ALD89130.1)	29.0%
**Order: *Mononegavirales* (Rhabdovirus-like)**							
Soybean thrips rhabdo-like virus 1	MT224147	10848	26,478	97%	556	Vesicular stomatitis Indiana virus (AYK03298.1)	33.0%
Soybean thrips rhabdo-like virus 2	MT224148	10807	19,259	97%	404	Vesicular stomatitis Indiana virus (AMK37545.1)	35.0%
Soybean thrips rhabdo-like virus 3	MW023861	12697	20,399	100%	368	Wuhan ant virus (YP_009304559.1)	32.8%
Soybean thrips negative-stranded RNA virus 4	MT224149	7486	1,350	60%	25	Hubei diptera virus 11 (YP_009337182.1)	39.5%
Soybean thrips negative-stranded RNA virus 5	MW033648	2500	443	20%	8	Gudgenby Calliphora mononega-like virus (QIJ70030.1)	32.1%
**Double-stranded RNA viruses**							
**Order:*Durnavirales* (Partitivirus-like)**							

Soybean thrips partiti-like virus 1	MT648422	1930	141	83%	15	Grapevine partitivirus (AFX73019.1)	52.2%
Soybean thrips partiti-like virus 2	MT648423	1520	1,202	100%	182	Hubei partiti-like virus 32 (APG78251.1)	56.3%
Soybean thrips partiti-like virus 3	MW039371	1523	37	64%	4	Rosellinia necatrix partitivirus (BBU59840.1)	53.6%
Soybean thrips partiti-like virus 4	MT648424	1470	476	100%	77	Hubei partiti-like virus 42 (APG78281.1)	56.7%
Soybean thrips partiti-like virus 5	MT648425	1375	3,267	98%	539	Hubei partiti-like virus 48 (APG78218.1)	53.0%
Soybean thrips partiti-like virus 6	MT648426	1377	2,504	100%	427	Osugoroshi virus 3 (BBE15518.1)	30.5%
Soybean thrips partiti-like virus 7	MT648427	1429	1,063	100%	174	Hubei partiti-like virus 48 (APG78218.1)	53.4%
Soybean thrips partiti-like virus 8	MT648428	2241	348	97%	17	Hubei partiti-like virus 23 (APG78232.1)	47.4%
Soybean thrips partiti-like virus 9	MT648429	1714	68,113	98%	9,128	Hubei diptera virus 18 (YP_009329892.1)	63.4%
Soybean thrips partiti-like virus 10	MT648430	1710	88	88%	11	Hubei partiti-like virus 17 (APG78256.1)	80.8%
Soybean thrips partiti-like virus 11, segment 1	MW023858	1613	546	90%	71	Hubei tetragnatha maxillosa virus 8 (YP_009337885.1)	57.6%
Soybean thrips partiti-like virus 11, segment 2	MW023859	1541	382	93%	50	Hubei tetragnatha maxillosa virus 8 (YP_009337888.1)	34.3%
Soybean thrips partiti-like virus 11, segment 3	MW023860	1482	366	92%	53	Hubei tetragnatha maxillosa virus 8 (YP_009337886.1)	28.4%
*Wuhan fly virus 5	MW039370	1617	461	97%	63	Wuhan fly virus 5 (YP_009342459.1)	93.9%
**Order: *Ghabrivirales* (Totivirus-like**)							
Soybean thrips-associated totivirus 1	MT293124	5662	1,281	100%	51	Umbelopsis ramanniana virus 2 (VFI65724.1)	44.6%
Soybean thrips-associated totivirus 2	MT293125	6778	4,164	88%	127	Hubei diptera virus 22 (YP_009336825.1)	37.2%
Soybean thrips-associated dsRNA virus 1	MT293146	3695	5,296	46%	149	Circulifer tenellus virus 1 (YP_003800003.1)	44.8%
Soybean thrips-associated dsRNA virus 2	MW039372	1479	51	19%	2	Scaphoideus titanus toti-like virus (QIJ56904.1)	31.4%
Soybean thrips-associated dsRNA virus 4	MW033658	3037	537	38%	14	Spissistilus festinus virus 1 (YP_003800000.1)	25.9%
**Order: *Reovirales* (Reovirus-like**)							
Soybean thrips-associated dsRNA virus 3, segment 1	MT648431	2410	228	58%	13	Shelly beach virus (AYP67577.1)	39.1%
Soybean thrips-associated dsRNA virus 3, segment 1	MW039372	1450	72	35%	4	Shelly beach virus (AYP67577.1)	42.2%
Soybean thrips-associated dsRNA virus 3, segment 4	MW039373	2471	179	69%	12	Shelly beach virus (AYP67580.1)	30.0%
**Single-stranded DNA viruses**							
**Order: *Piccovirales* (Densovirus-like)**							
Soybean thrips denso-like virus 1	MT240783	3829	788	97%	37	Aedes albopictus densovirus (AYH52678.1)	36.9%
Soybean thrips denso-like virus 2	MW039377	3403	188	68%	5	Viltain virus (AQN78648.1)	38.6%

* previously described viruses detected in this study.
# plant viruses detected in this study.
+ viruses that infect plants and thrips.
